# Focused Cardiac Ultrasound Examination as a Tool for Diagnosis of Infective Endocarditis and Myocarditis in Dogs and Cats

**DOI:** 10.3390/ani11113162

**Published:** 2021-11-05

**Authors:** Olga Szaluś-Jordanow, Marta Stabińska-Smolarz, Michał Czopowicz, Agata Moroz, Marcin Mickiewicz, Andrzej Łobaczewski, Dorota Chrobak-Chmiel, Magdalena Kizerwetter-Świda, Magdalena Rzewuska, Rafał Sapierzyński, Michał Grzegorczyk, Anna Świerk, Tadeusz Frymus

**Affiliations:** 1 Department of Small Animal Diseases with Clinic, Institute of Veterinary Medicine, Warsaw University of Life Sciences-SGGW, Nowoursynowska 159 Street, 02-776 Warsaw, Poland; tadeusz_frymus@sggw.edu.pl; 2 Private Practice, Wyszyńskiego 12/11 Street, 41-200 Sosnowiec, Poland; marta_stabinska@yahoo.co.uk; 3 Division of Veterinary Epidemiology and Economics, Institute of Veterinary Medicine, Warsaw University of Life Sciences-SGGW, Nowoursynowska 159 Street, 02-776 Warsaw, Poland; michal_czopowicz@sggw.edu.pl (M.C.); agata_moroz@sggw.edu.pl (A.M.); marcin_mickiewicz@sggw.edu.pl (M.M.); 4 Round-the-Clock Veterinary Clinic Auxilium, Królewska Street 64, 05-822 Milanówek, Poland; alobaczewski007@gmail.com; 5 Department of Preclinical Sciences, Institute of Veterinary Medicine, Warsaw University of Life Sciences-SGGW, Nowoursynowska 159 Street, 02-776 Warsaw, Poland; dorota_chrobak@sggw.edu.pl (D.C.-C.); magdalena_kizerwetter_swida@sggw.edu.pl (M.K.-Ś.); magdalena_rzewuska@sggw.edu.pl (M.R.); 6 Department of Pathology and Veterinary Diagnostic, Institute of Veterinary Medicine, Warsaw University of Life Sciences-SGGW, Nowoursynowska 159 Street, 02-776 Warsaw, Poland; rafal_sapierzynski@sggw.edu.pl; 7 Department of Descriptive and Clinical Anatomy, Medical University of Warsaw, Chałbińskiego 5 Street, 02-004 Warsaw, Poland; grzegorczyk.m.s@gmail.com; 8 Round-the-Clock Veterinary Clinic LEGWET, Jagiellońska 20, 05-120 Legionowo, Poland; kornatowska.an@gmail.com

**Keywords:** infective endocarditis, myocarditis, dogs, cats

## Abstract

**Simple Summary:**

Infective endocarditis and myocarditis are rarely diagnosed conditions with high mortality rates in dogs and cats. As their clinical signs are highly nonspecific, and advanced cardiological diagnostics are not always available in small animal clinics, both diseases seem to be underdiagnosed. Infective endocarditis affects mainly the mitral and aortic valves. In myocarditis, thickening of the left ventricle walls is usually observed; however, in some cases dilatation of the left ventricle lumen with thinning of the walls can be visible. In human medicine, basic cardiac ultrasound examination enables a quick initial diagnosis of these two conditions. Using this technique, we tentatively diagnosed 7 cases of myocarditis and/or infective endocarditis in dogs and cats, and these diagnoses were later confirmed by other methods. Because the initial diagnoses were made by a general practitioner after relatively short training, not by a cardiology specialist, we conclude that common use of this technique in small animal practices equipped with ultrasound device could increase the rate of diagnosis of these conditions, leading to earlier treatment initiation.

**Abstract:**

Symptoms of infective endocarditis (IE) and myocarditis are usually nonspecific and include fever, apathy, and loss of appetite. This condition can lead to severe heart failure with ascites or/and fluid in the thoracic cavity or/and in the pericardial sac. We describe infective endocarditis and myocarditis in 3 dogs and 4 cats. In all animals, the initial diagnosis was performed on the basis of a focused cardiac ultrasound examination performed by a general practitioner after a training in this technique. The initial findings were confirmed by a board-certified specialist in veterinary cardiology. Post mortem positive microbiological results from valves were obtained in 4 of 7 patients. Methicillin-resistant *Staphylococcus aureus* was confirmed in 2 cases and *Staphylococcus epidermidis* was confirmed in 2 cases, one of which included *Enterococcus* sp. coinfection. Histopathological examination confirmed initial diagnosis in 5 of 7 animals. In the remaining 2 patients, the time elapsed from the onset of clinical symptoms to death was about 1 month and no active inflammation but massive fibrosis was found microscopically. This is, to our best knowledge, the first report of IE and myocarditis diagnosed in small animals using focused cardiac ultrasound examination. Therefore, we conclude that common usage of this technique by trained general veterinarians may increase the rate of diagnosed patients with these conditions.

## 1. Introduction

Infective endocarditis (IE) is an uncommon condition of the endothelium of the valves, ventricles, atria, or large blood vessels [1]. Most frequently the aortic and mitral valves are involved in dogs and cats. IE is more common in middle-sized dogs than it is in large breed dogs [2], while in cats this disease is extremely rarely diagnosed [3]. Congenital heart disease, especially subaortic stenosis, and other structural heart diseases are considered risk factors for IE [4]. Myocarditis is an inflammation of the heart muscle characterized by infiltration of myocardial tissue by inflammatory cells [1]. Generally, myocarditis may develop in two forms: one with thickening of the ventricular walls, and one with ventricular lumen dilation. Disturbances in the movement of the heart walls can be also visible, and in some cases fluid in the pericardial sac can be found. Echocardiography is a basic, non-invasive diagnostic tool that is also important in monitoring of heart changes in the course of these diseases. However, an ultrasound of the heart is usually not helpful in determining the etiology of IE or myocarditis. In human medicine, myocarditis is divided into two clinical forms: fulminant and acute. Patients with fulminant myocarditis have a nondilated, thickened and hypocontractile left ventricle (LV), whereas patients with acute disease have significantly dilated left ventricular lumen, normal or thin walls, and severely impaired contractility [5]. Depending on the form of myocarditis, echocardiography shows global or regional LV dysfunction as manifested by reduced ejection fraction, disturbance in the wall motion and enlargement of its lumen or transient symmetric or asymmetric wall thickening. In human medicine myocarditis with dilation is more common than with hypertrophy [5,6,7,8]. In dogs, as in humans, the clinical signs of IE and myocarditis are nonspecific and include high fever or prolonged sub-febrile states, weakness, sometimes accompanied by respiratory distress, and thromboembolic problems which makes the diagnosis challenging. In dogs and cats, respiratory signs and locomotory abnormalities of varying severity can also be observed [9,10].

Both conditions are probably underdiagnosed in animals due to nonspecific clinical signs and diagnostic difficulties. The diagnosis of IE usually relies on blood cultures and, less commonly, on molecular biology techniques such as polymerase chain reaction (PCR) or matrix-assisted laser desorption/ionization–time of flight (MALDI-TOF). In human medicine, microbiological and histopathological examination of the removed valve is typically performed after a surgery. In animals, such an examination may only be performed after death [11,12]. In antemortem diagnosis, echocardiography is very useful in both human and veterinary medicine. Usually, a definitive diagnosis of IE can be attained by fulfilling two major criteria, referred to as Duke’s criteria, which are a positive blood culture and echocardiographic evidence of vegetative lesions on cardiac valves [13,14]. However, blood cultures have been found diagnostic in less than 60% cases, which could result from the fact that 3/4 of negative culture patients received antibiotics prior to blood culturing [15]. In veterinary medicine, traditional blood culture has been shown to yield positive results in roughly 40% cases [16]. This makes echocardiography an important independent modality in antemortem diagnosis [17].

The definitive diagnosis of myocarditis relies on a myocardial biopsy, which even in humans is performed rarely in everyday practice, or in postmortem histopathological examination. Before the introduction of echocardiography, IE and myocarditis were usually recognized during surgery or autopsy [18]. In the past, human IE and myocarditis were rarely recognized in internal medicine departments, as the specialists capable of performing reliable echocardiographic examination were usually lacking. This resulted in a significant delay in proper diagnosis and treatment. After the introduction of the so-called focused cardiac ultrasound examination (known also as bedside emergency cardiac ultrasound) performed by trained emergency physicians, the diagnosis of IE and myocarditis is made sooner, leading to more effective treatment [19,20,21,22].

Because echocardiography has recently become a widely available diagnostic modality in veterinary medicine, we decided to analyze the usefulness of focused cardiac ultrasound in the diagnosis of IE and myocarditis in dogs and cats.

## 2. Materials and Methods

### 2.1. The Persons Performing the Heart Ultrasound Examination

Focused cardiac ultrasound examinations were performed by a general practitioner after 8 h of training in this technique. In each case, a full echocardiography examination was also performed by a board-certified specialist with 14 years of experience in echocardiography. The time between the fast and the full echocardiographic examination ranged from 10 min to 6 h, depending on the case.

### 2.2. Ultrasound Device

Focused cardiac ultrasound examination was performed using a GE Healthcare Logiq F6 (Chicago, WI 53188, USA) ultrasound device with a 10-6 MHz microconvex transducer both for dogs and cats. Specialist echocardiography in cases 2 to 6 was performed using a Mindray M7 with a 4-2s MHz phased array transducer in dogs and a 12-4s MHz phased array transducer (Shenzhen 518057, China) in cats. In cases 1 and 7, a Mindray M9 (Shenzhen 518057, China) with a 5-1s MHz phased array transducer in dogs and 10-4s MHz phased array transducer in cats were used.

### 2.3. Animals

The study enrolled only these dogs and cats suspected of IE or myocarditis that died or were euthanized. Seven animals initially diagnosed with IE and/or myocarditis using focused cardiac ultrasound examination by a general practitioner were re-examined to confirm the initial findings. All patients were referred for an urgent examination to a board-certified ultrasound specialist who confirmed valve vegetation or thickening of heart muscle. This examination was performed within 10 min to 6 h from the focused heart ultrasound examination. All 7 animals died or were euthanized, and myocardial and valvular tissue samples were collected post mortem for histopathological and microbiological examination. Samples for histopathology were fixed in 4% formalin, stained with hematoxylin and eosin, and 4-µm thick slides were examined under a light microscope. Culturing of blood or tissue samples was performed by routine procedures used for the detection of aerobic and anaerobic mesophilic bacteria. All isolates were identified using a VITEK 2-compact automated system (bioMérieux, Craponne, France). In some cases, pulse field gel electrophoresis (PFGE) was used for the determination of a genetic relationship of isolated bacteria according to Murchan et al. [23]. All patients suspected of IE and/or myocarditis in which the implemented antibiotic treatment resulted in clinical improvement were excluded from the study.

#### 2.3.1. Case No. 1

**Figure 1:**
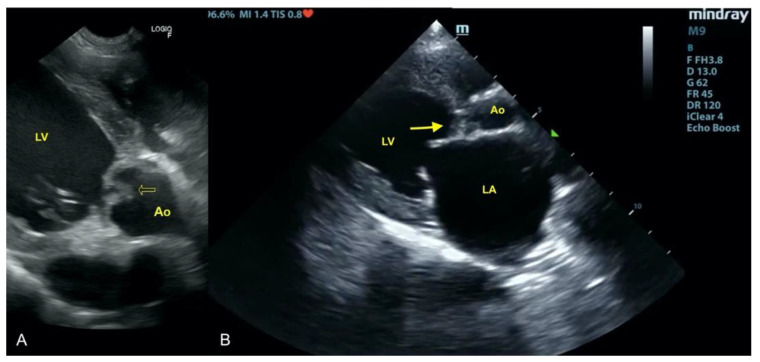
Vegetations and proliferative lesions on the aortic valve. Ao—aorta, LV—left ventricle LA—left atrium, yellow arrow—changed aortic leaflets. (**A**) Scan from microconvex probe obtained during focused cardiac ultrasound examination (GE Healthcare Logiq F6). (**B**) Scan from phased array probe obtained during echocardiography examination performed by a board-certified echocardiography specialist (Mindray M9).

A 12 year-old bitch was referred to the clinic with high fever (up to 41 °C) lasting over two weeks, with no appetite. On admission, the dog was unconscious and showed dyspnea (50 breaths per minute but during hospitalization increased to 65). The history revealed that 3 weeks earlier the dog had undergone a mastectomy due to necrotic mammary gland tumor. Fever emerged four days after the surgery. Temporary improvement was observed following antimicrobial (ceftiofur, metronidazole) and anti-inflammatory (meloxicam) treatment. However, the dog’s condition deteriorated, and the patient was presented to our clinic. A blood check-up revealed leukocytosis 29 G/L (mean 6–12), elevated activity of liver enzymes- alanine aminotransferase (ALT) 250 U/I (30–60); aspartate aminotransferase (AST) 130 (1–45), prolonged prothrombin time (14 s (mean 7–10), and decreased fibrinogen concentration (0.5 g/L (mean 1–5) which suggested disseminated intravascular coagulation (DIC). Focused ultrasound examination showed vegetation on the aortic valve up to 1 cm in diameter. The valve was thickened and did not fully open (Figure 1). A specialized echocardiographic examination performed 6 h later confirmed the suspected aortic stenosis with a blood flow of 4.5 m/s. Blood was collected for microbiological examination. As the patient’s condition was not improving and the prognosis was grave, the owners decided to euthanize the dog a few hours later. During autopsy, severe thickening with advanced productive lesions of all three aortic leaflets was detected.

#### 2.3.2. Case No. 2

**Figure 2:**
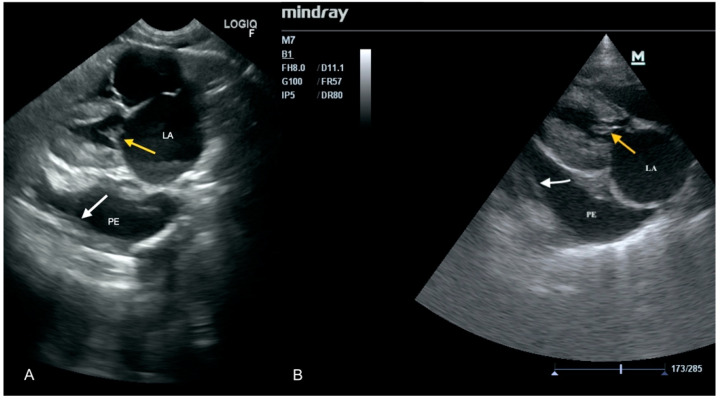
Vegetations on the anterior leaflet of the mitral valve (yellow arrow), enlarged left atrium (LA), and pericardial effusion (PE); the white arrow points to the percicardial sac. (**A**) Scan from a microconvex probe obtained during focused cardiac ultrasound examination (GE Healthcare Logiq F6). (**B**) Scan from a phased array probe obtained during echocardiography examination performed by a board-certified echocardiography specialist (Mindray M7).

A 2 year-old queen was referred to the clinic being unconscious and showing dyspnea (70 breaths per minute). According to the owners, the cat had poor appetite and had been lethargic for a few days. Dyspnea appeared only a few hours before the admission. A global focused assessment sonography for trauma (FAST) was performed, which included a short echocardiography, and chest and abdominal ultrasound examination. Echocardiography showed vegetation on both the aortic and mitral valves, and a large amount of effusion in the pericardial sac, causing a cardiac tamponade (Figure 2). In the pleural and peritoneal cavities, a large amount of free fluid was found. Basing on these findings, the initial diagnosis was IE and myocarditis. These findings were confirmed in a specialist echocardiographic examination performed 10 min later. Oxygen therapy was started, and an intravenous catheter was established. The cat was prepared for puncture of the pericardial sac. Unfortunately, before the pericardiocentesis was initiated, a respiratory and cardiac arrest appeared, and resuscitation failed. During autopsy, large amounts of fluid in pericardial sac, thickening of left ventricle walls and thickening of the aortic and mitral valves were confirmed.

#### 2.3.3. Case No. 3

**Figure 3:**
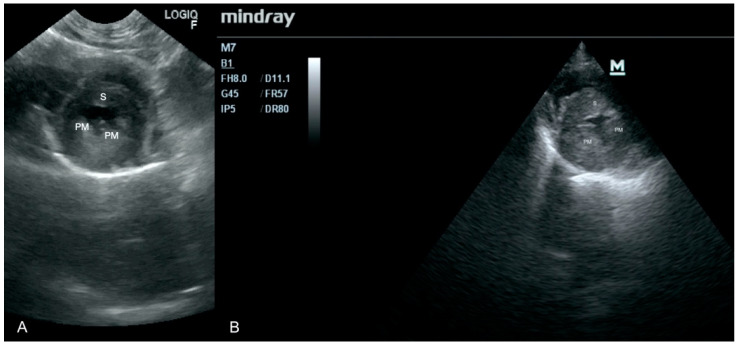
Thickening of left ventricle walls. PM—papillary muscle, S—septum. (**A**) Scan from a microconvex probe obtained during focused cardiac ultrasound examination (GE Healthcare Logiq F6). (**B**) Scan from phased array probe obtained during echocardiography examination performed by a board-certified echocardiography specialist (Mindray M7).

An 8 month-old castrated, outdoor male cat was referred to the clinic in a bad general state. It had a small wound on a limb and was treated with amoxicillin with clavulanic acid for 2 days before high fever (41 °C) emerged and its general condition suddenly worsened. Focused ultrasound examination of the heart showed thickening of the valves and thickening of the left ventricular muscle. These findings were confirmed by a specialist 5 h later (Figure 3). After this examination, the owners decided to euthanize the animal due to bad prognosis and lack of improvement after treatment. On necropsy, significant thickening of the left ventricle wall and numerous whitish foci on both ventricles were found.

#### 2.3.4. Case No. 4

**Figure 4:**
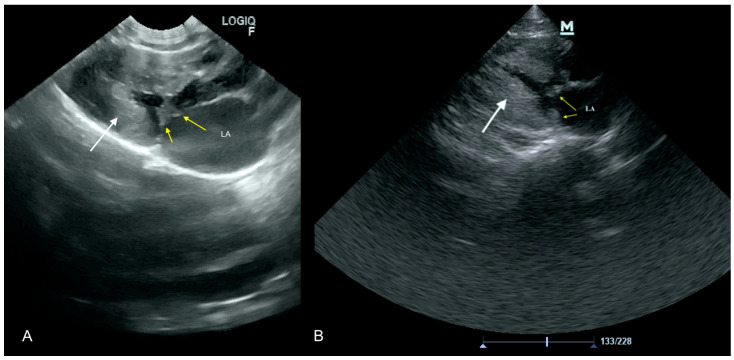
Yellow arrows—thickening of the mitral valve leaflets, white arrow—thickening of left ventricle free wall, LA—left atrium. (**A**) Scan from a microconvex probe obtained during focused cardiac ultrasound examination (GE Healthcare Logiq F6). (**B**) Scan from a phased array probe obtained during echocardiography examination performed by a board-certified echocardiography specialist (Mindray M7).

An 8-year-old castrated male cat was hospitalized due to apathy and lack of appetite for 3 days. A blood check-up showed significant leukocytosis 45 G/L (mean 6–12) and the abdominal ultrasound examination revealed massive ascites. As inflammation of unknown origin was suspected, the cat was treated with amoxicillin and furosemide because of the ascites. However, its clinical condition did not improve significantly. Two weeks later a heart murmur appeared, and a focused echocardiographic examination was performed, which revealed thickening of the valvular leaflets and thickening of left ventricle walls. An initial diagnosis of IE was made. A specialist echocardiographic examination performed 6 h later confirmed this finding (Figure 4) and also revealed mitral and tricuspid regurgitation. The previously used antibiotics were completed and ceftriaxone (30 mg per kg twice daily) was introduced. Slight clinical improvement was observed but the leukocytosis 47 G/L (mean 6–12) persisted. Two weeks after echocardiography, the owner decided to euthanize the animal due to clinical deterioration and severe dyspnea. During autopsy, numerous purulent lesions in the lungs, vegetation on the mitral and tricuspid valves, significant enlargement of both atria and thickening of the left ventricle walls were detected.

#### 2.3.5. Case No. 5

**Figure 5:**
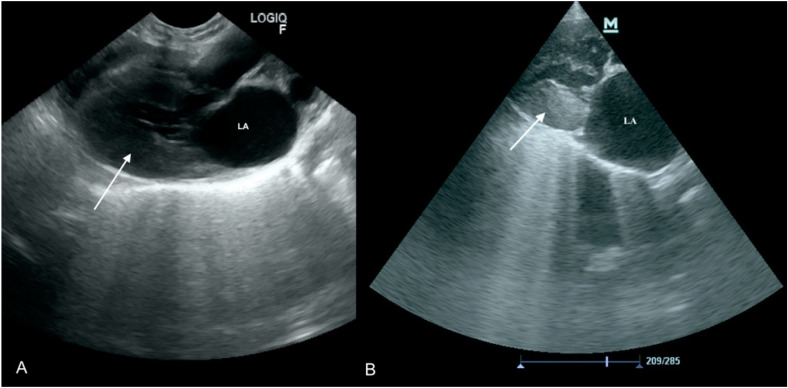
(**A**) White arrow points to the thickened left ventricle free wall, LA—left atrium. (**A**) Scan from a microconvex probe obtained during focused cardiac ultrasound examination GE (Healthcare Logiq F6). (**B**) Scan from a phased array probe obtained during echocardiography examination performed by a board-certified echocardiography specialist (Mindray M7).

An 8-year-old Yorkshire Terrier was referred to the clinic because of coughing and reduced exercise tolerance. Echocardiography showed a thickening of the mitral valve leaflets and a moderate left atrial enlargement. Degenerative mitral valve disease (DMVD) with secondary enlargement of the left atrium was diagnosed. Benazepril and furosemide were applied, and the dog’s condition slightly improved. After a month, the patient was presented to the clinic again, lethargic, with mild dyspnea (43 breaths per min). The focused echocardiographic examination revealed significant thickening of the mitral valve but this time the aortic valve was also very significantly thickened, as were the left ventricle walls. After the patient’s condition was stabilized using oxygen therapy and high doses of furosemide, which took 6 h, a full cardiological examination confirmed the previous lesions and visualized the aortic (4.5 m/s), pulmonary (3.1 m/s), and mitral valve (2.6 m/s) stenosis with significant asymmetric thickening of left ventricle walls (Figure 5). As fever was absent, antibiotic therapy was not introduced. The patient was referred for a blood culture on the next day due to suspected myocarditis and IE based on aortic valve and left ventricle thickening not observed in first echocardiography examination. Unfortunately, the dog died during the night. Autopsy revealed lesions on the mitral and aortic valves with significant thickening of the left ventricle walls.

#### 2.3.6. Case No. 6

**Figure 6:**
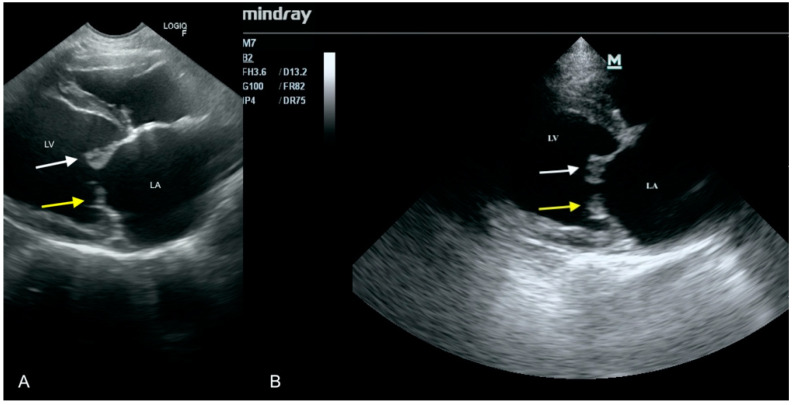
White arrow points to advanced vegetation on the anterior leaflet of mitral valve; yellow arrow points to advanced vegetation on the posterior leaflet of mitral valve. LA—left atrium, LV—left ventricle. (**A**) Scan from a microconvex probe obtained during focused cardiac ultrasound examination (GE Healthcare Logiq F6). (**B**) Scan from phased array probe obtained during echocardiography examination performed by a board-certified echocardiography specialist(Mindray M7).

A 12 year-old bitch was referred to the clinic due to reduced appetite. Clinical examination revealed high fever (40 °C), with no other clinical signs. The dog was admitted to the hospital and a blood check-up was performed leukocytes 38 G/L (mean 6–12). Treatment with antibiotics was introduced (amoxicillin 7 mg/kg with clavulanic acid 1.75 mg/kg once daily subcutaneous (S.C.) injection). Since the manual blood smear revealed morulae, indicating *Ehrlichia* spp. infection, doxycycline 10 mg/kg per os (P.O.) once daily was added to the treatment. After a temporary improvement, the dog returned a few days later to the clinic in poor general state. High-fever (40.5 °C) and dyspnea (60 breaths per minute) were present. This time a heart murmur was found on auscultation, which was not detected during the previous visits. Focused ultrasound examination showed vegetation (size 0.5 cm × 0.7 cm) on the mitral valve and the left atrium and left ventricle enlargement. These findings were confirmed in a specialist echocardiography examination (Figure 6) performed 4 h later, which also revealed massive mitral and aortic valve insufficiency. On this basis, IE accompanied by the congestive heart failure was suspected. Pimobendan (0.3 mg/kg twice daily P.O.) and enrofloxacin (5 mg/kg S.C. once daily) treatment led to a temporary improvement. However, in a short time the patient’s condition worsened and the owner decided to perform euthanasia. Autopsy revealed severe thickening of mitral valve with enlargement of left ventricle and left atrium.

#### 2.3.7. Case No. 7

**Figure 7:**
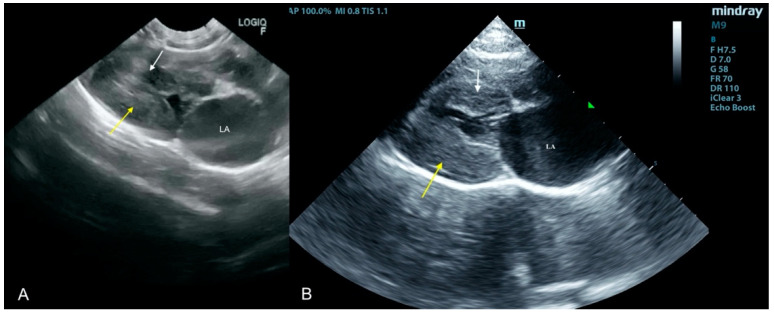
Thickening of left ventricle walls. LA—left atrium, yellow arrow points to the free wall of the left ventricle, white arrow points to the septum. (**A**) Scan from a microconvex probe obtained during focused cardiac ultrasound examination (GE Healthcare Logiq F6). (**B**) Scan from a phased array probe obtained during echocardiography examination performed by a board-certified echocardiography specialist (Mindray M9).

A 1-year-old male cat was admitted to the clinic due to apathy and lack of appetite. The cat had a fever (up to 41 °C). Amoxicillin with clavulanic acid (amoxicillin 7 mg/kg with clavulanic acid 1.75 mg/kg once daily S.C. injection) and tolfenamic acid (4 mg/kg S.C. injection once daily) were administered. Within 24 h, there was a significant deterioration, with increased breathing of up to 40 breaths per minute. The ultrasound examination of the heart revealed a considerable thickening of the heart muscle, with enlargement of the left atrium. The results of the specialist echocardiography examination were consistent with the focused heart examination. The time between both exams was 2.5 h (Figure 7). Considering the clinical condition and both echocardiography exams, myocarditis was suspected. Blood was collected for microbiological examination, and third-generation cephalosporin (cefovecin sodium 8 mg/kg S.C. injection) and marbofloxacin (5 mg/kg P.O. once daily) were introduced for treatment. Unfortunately, the cat’s condition deteriorated gradually over the next 24 h, leading to respiratory and cardiac arrest. The autopsy revealed severe thickening of left ventricle walls with left atrium enlargement.

## 3. Results

### 3.1. Case No. 1

**Figure 8:**
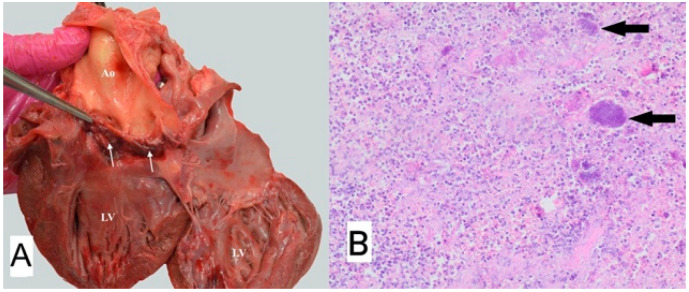
(**A**) Macroscopic view of vegetation of the aortic valve. White arrow points to thickened aortic valve leaflets with massive proliferative lesions, Ao—Aorta, LV—left ventricle. (**B**) Microscopic view of sample–necrotic and purulent inflammation with numerous bacterial colonies (arrows); H-E, magnification 100×.

During the autopsy, a swab was taken for microbiological examinations from the aortic valve, and the valve itself was taken for histopathological examination. Microscopically purulent endocarditis with numerous bacterial colonies covering endocardial surface were found (Figure 8). Methicillin-resistant *Staphylococcus aureus* (MRSA) was isolated from both the blood taken antemortem and the valvular sample collected during autopsy. Pulsed-field gel electrophoresis (PFGE) confirmed that both isolates were genetically identical and belonged to the same PFGE type.

### 3.2. Case No. 2

**Figure 9:**
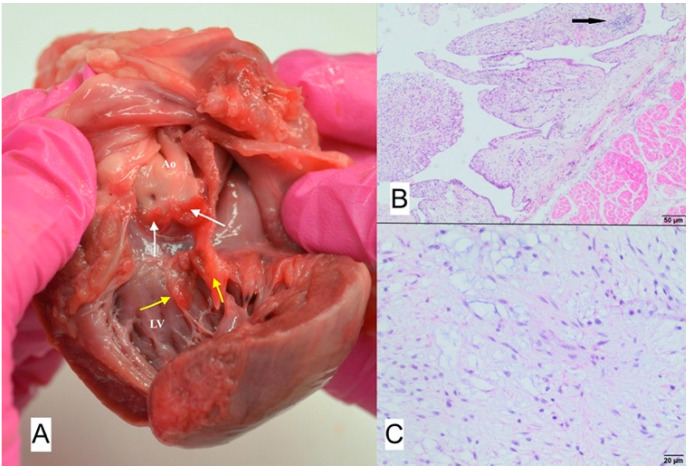
(**A**) Macroscopic view of thickened aortic and mitral valves. White arrows point to the aortic valve leaflets, yellow arrows points to the mitral valve, Ao—Aorta, LV—left ventricle. (**B**) Microscopic view: myocarditis with multifocal necrosis of cardiomyocytes, and moderate inflammatory infiltrate consisting of histiocytes and lymphocytes; H-E; magnification. 100× and proliferative pericarditis end endocarditis with mild multifocal lymphocytic inflammatory infiltrate and proliferation of fibrous tissue with maturation to muco-fibroid tissue in subserosal layer of serosal membranes. Exophytic proliferation of fibrous tissue covered by mesothelium with sparse lymphoid inflammatory infiltrate–arrow; H-E, magnification. 4×. (**C**) Close view of mature connective tissue with accumulation of muco-fibrous matrix; H-E, magnification 100×.

During the autopsy, a swab was taken for microbiological examinations from the aortic and mitral valves, and the valves and left ventricle muscle were taken for histopathological examinations. Methicillin-susceptible *Staphylococcus epidermidis* and *Enterococcus* sp. were isolated from the aortic and mitral valve lesions. Multifocal myocarditis with mixed histiocytic and lymphocytic infiltration, proliferative pericarditis and endocarditis were histopathologically found (Figure 9).

### 3.3. Case No. 3

**Figure 10:**
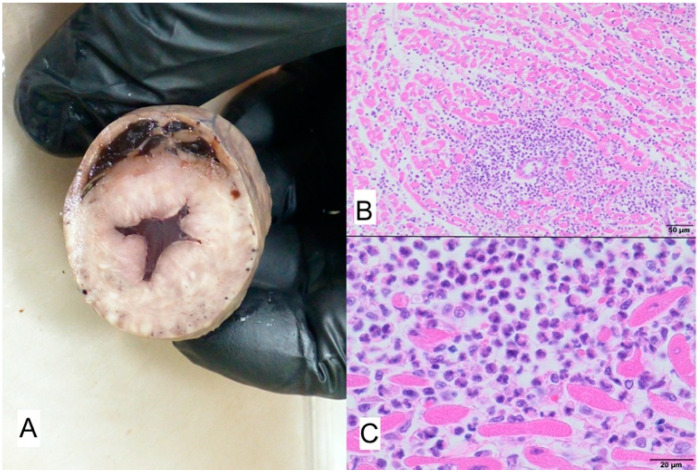
(**A**) Macroscopic view of transverse section of feline heart (specimen fixed in formalin)—numerous visible, randomly distributed, poorly demarcated, pale foci of inflammation; visible transverse section of blood vessels seen at the periphery of left ventricular wall. Microscopic view: (**B**) focal (perivascular) to diffuse inflammatory infiltrate between cardiomyocytes, Hematoxylin-eosin, magnification 40×. (**C**) Consisting mainly of neutrophils with few macrophages and magnification 400×.

During the autopsy, part of the left ventricle muscle was taken for microbiological examinations and the left ventricle muscle was taken for histopathological examinations. During histopathological examination, acute perivascular to diffuse purulent myocarditis was detected (Figure 10). Culturing of the heart muscle specimens failed to detect bacteria or fungi.

### 3.4. Case No. 4

**Figure 11:**
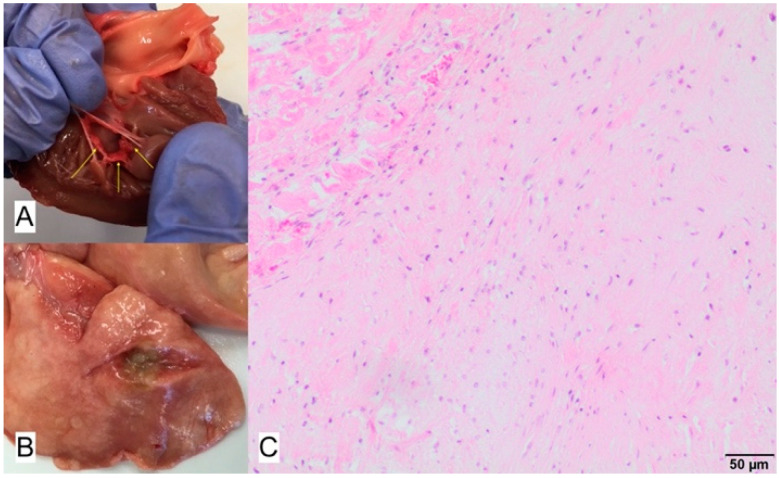
(**A**) Macroscopic view: yellow arrows point to the thickened mitral valve. (**B**) Macroscopic view: large number of purulent lesions in the lungs (**C**). Microscopic view of base of the valve presenting increased amount of fibrous extracellular matrix; hematoxylin-eosin, magnification 40×.

During the autopsy, a swab was taken for microbiological examinations from the valves and lung, and the same material was taken for histopathological examinations. Histopathological examination revealed multifocal cardiomyocyte hypertrophy with disarrangement, and multifocal interstitial fibrosis in the heart muscle. In addition, an increased amount of fibroid matrix in the valvular stroma was observed (Figure 11). Microbiological examination of swabs taken from lung lesion and the mitral and tricuspid valves did not reveal bacteria or fungi.

### 3.5. Case No. 5

**Figure 12:**
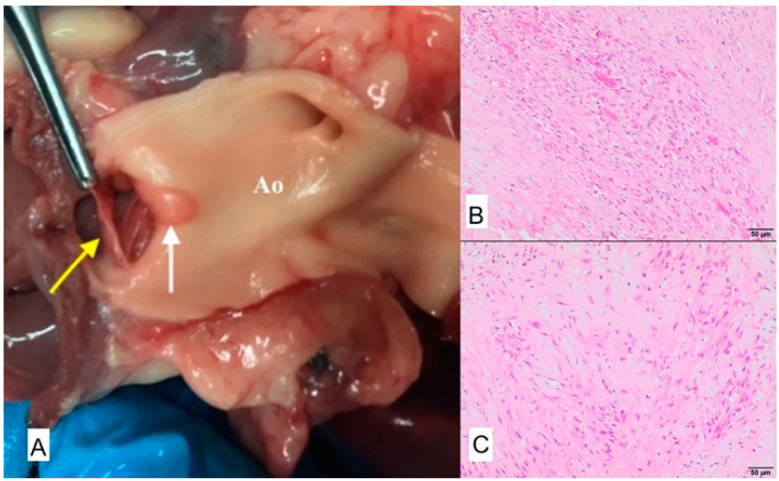
(**A**) Macroscopic view of aorta. Yellow arrow poins to the thicked aortic valve leaflet, white arrow poitns to the proliferative lesion on the aortic wall, Ao—Aorta. Microscopic view: chronic endocartitis with massive valvular fibrosis. (**B**) Fibrovascular tissue of aortal valve; H-E, magnification 100×. (**C**) Fibrous connective tissue of mitral valve; H-E, magnification 100×).

During the autopsy, a swab was taken for microbiological examinations from the aortic valve and the valve itself was taken for histopathological examinations. Microscopically chronic endocarditis with massive valvular fibrosis affecting the valves was observed (Figure 12). No bacteria or fungi were detected after culturing.

### 3.6. Case No. 6

**Figure 13:**
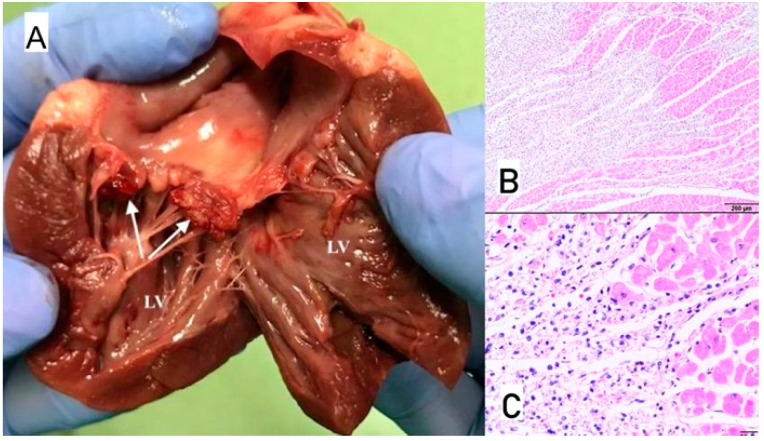
(**A**) Macroscopic view: white arrows point to the massive vegetations on mitral valve, LV—left ventricle. (**B**) Inflammatory infiltrate of myocardium; H-E, magnification 40×. (**C**) Higher magnification of inflamed area: infiltration with mononuclear cells is visible; H-E, magnification 100×.

During the autopsy, a swab was taken for microbiological examinations from the mitral valve and valve itself and part of left ventricle muscle for histopathological examinations. Histopathological examination revealed multifocal myocarditis with mixed, predominantly mononuclear inflammatory cellular infiltrate, areas of myocardial necrosis, and endocarditis with fibrin deposits on their surface. (Figure 13) Methicillin-resistant *S. epidermidis* was cultured from the valvular vegetations after post-mortem examination.

### 3.7. Case No. 7

**Figure 14:**
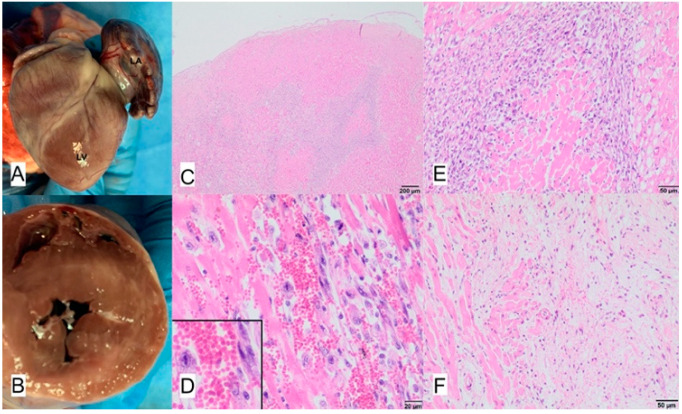
(**A**) Massive enlargement of left atrium, LA—left atrium, LV—left ventricle. (**B**) Thickening of myocardium of left ventricle with numerous pale areas. (**C**) Histopathologic view of myocardium and base of the mitral valve, (**D**) consisting of mononuclear cells, mainly macrophages. (**E**) inflammatory infiltrate producing coalescing nests and bands surrounding cardiomyocytes. (**F**) Macrophages resembling Anitschkow cells (inlet), penetrating the base of mitral valve (border between myocardium, on the left; base of mitral valve, on the right); hematoxylin-eosin stain, magnification bar on particular figures.

During the autopsy, a swab was taken for microbiological examinations from the mitral valve and part of left ventricle muscle. For histopathological examinations, the left ventricle muscle and mitral valve were taken. Multifocal mixed inflammation of the myocardium with histiocyte and lymphocyte infiltration and proliferation of interstitial fibrous connective tissue were detected during microscopic examination. Diffuse inflammatory infiltrate was also present in the valvular stroma (Figure 14). Methicillin-resistant *S. aureus* was cultured ante mortem from the blood and post mortem from the valves and heart muscle.

## 4. Discussion

In the IE and/or myocarditis cases presented in this paper the preliminary diagnosis made by a general practitioner after a short training was confirmed by both a board-certified ultrasound specialist and by post mortem examination (histopathology and/or microbiology). These results suggest that use of focused cardiac ultrasound examination in the routine veterinary practice could increase the frequency of diagnosis of IE or myocarditis in dogs and cats. Each practitioner with an ultrasound device can easy acquire the needed skills, and this does not require a long training time. In addition, in the vast majority of cases, such examination can be performed with a convex probe available in most ultrasound devices. In human medicine, such an emergency ultrasound examination is now performed routinely. Depending on the author, it is called bedside ultrasound [19], focused cardiac ultrasound [20], or point-of-care ultrasound (POCUS) [24]. While full echocardiography is traditionally performed in cardiology departments, emergency ultrasound allows an earlier diagnosis on admission [19]. This, in turn, enables an earlier initiation of treatment, which considerably improves prognosis.

Even though echocardiography has been performed in animals for over 40 years, the patients described in this study are to our best knowledge the first IE and myocarditis animal cases diagnosed using focused cardiac ultrasound examination. This likely results from non-specific clinical signs which do not lead to a cardiological consultation, and such cases can remain underdiagnosed. Therefore, more common focused echo ultrasound examination performed by trained general practitioners are likely to increase the frequency of these diagnoses.

In 4 of 7 patients (57%) the infectious agent was cultured from the valves or heart muscle collected in an autopsy. The percentage of positive valve cultures was higher than reported in human studies when the material came from surgically replaced valves. Munoz et al. detected an infectious agent in 25% of patients, while Brandao at al. detected them in 15%, and in Greub et al., only 13% [15,25,26]. Culturing results have been shown to be negatively linked with the duration of antimicrobial treatment [27]. The situation probably looks similar in veterinary medicine. In our study, one negative valvular culture referred to a cat (case no. 4) that was treated with antibiotics for almost a month. In another patient (case no. 3) with histopathological confirmation of myocarditis, microbiological examination was negative. This is consistent with the observation that some bacterial agents cannot be detected in routine microbial cultures or can belong to culture-negative types such as *Bartonella* sp. [16,28]. The other possible explanation of negative cultures is the rheumatic myocarditis/endocarditis. In humans, this disease is initiated by a bacterial infection (mainly *Streptococcus pyogenes*) followed by an autoimmune reaction resulting in valve thickening and fibrosis. This could have also been the case in both our patients without inflammatory infiltration on the valves or heart muscle (case no. 4 and 5) [29]. In the remaining two animals with no inflammatory infiltration, fibrous tissue was present, which could suggest a subsiding phase of inflammation in these patients, especially given the one-month period which had elapsed from the onset of first symptoms to death. The absence of inflammatory changes on the valves and the heart muscle is consistent with the observations from human medicine that both valve necrosis and acute inflammation decrease with time in the course of the disease. After a longer time, only post-inflammatory fibrosis is observed, without the presence of active inflammatory infiltration [30]. This situation was observed in the patient no. 5, who presented with the appearance of advanced lesions initially on the mitral valve, and then one month later lesions were observed on the aortic valve, causing severe aortic stenosis before, finally, thickening of the heart muscle was observed. Our result is consistent with a recent human study, where histopathological examination of surgically removed valves with diagnosis based on Duke’s criteria confirmed IE in 80% of cases [15].

## 5. Conclusions

Focused cardiac ultrasound examination performed by a general small animal practitioner after a short training can be used as tool for the antemortem detection of infective endocarditis and myocarditis. As in human medicine, this procedure, performed as a part of the initial patient assessment, enables earlier treatment. It can also be the basis for referral to an urgent cardiology specialist consultation.

## Data Availability

The data sets used and/or analyzed are available from the corresponding author on reasonable request.
